# Machine learning-optimized Combinatorial MRI scale (COMRISv2) correlates highly with cognitive and physical disability scales in Multiple Sclerosis patients

**DOI:** 10.3389/fradi.2022.1026442

**Published:** 2022-11-11

**Authors:** Erin Kelly, Mihael Varosanec, Peter Kosa, Vesna Prchkovska, David Moreno-Dominguez, Bibiana Bielekova

**Affiliations:** ^1^ Neuroimmunological Diseases Section, Laboratory of Clinical Immunology and Microbiology, National Institute of Allergy and Infectious Diseases, National Institutes of Health, Bethesda, MD, United States; ^2^ QMENTA, Boston,MA, United States

**Keywords:** machine learning (ML), multiple sclerosis, MRI biomarkers, disability outcomes, predictive models

## Abstract

Composite MRI scales of central nervous system tissue destruction correlate stronger with clinical outcomes than their individual components in multiple sclerosis (MS) patients. Using machine learning (ML), we previously developed Combinatorial MRI scale (COMRISv1) solely from semi-quantitative (semi-qMRI) biomarkers. Here, we asked how much better COMRISv2 might become with the inclusion of quantitative (qMRI) volumetric features and employment of more powerful ML algorithm. The prospectively acquired MS patients, divided into training (*n* = 172) and validation (*n* = 83) cohorts underwent brain MRI imaging and clinical evaluation. Neurological examination was transcribed to NeurEx™ App that automatically computes disability scales. qMRI features were computed by lesion-TOADS algorithm. Modified random forest pipeline selected biomarkers for optimal model(s) in the training cohort. COMRISv2 models validated moderate correlation with cognitive disability [Spearman Rho = 0.674; Lin's concordance coefficient (CCC) = 0.458; *p* < 0.001] and strong correlations with physical disability (Spearman Rho = 0.830–0.852; CCC = 0.789–0.823; *p* < 0.001). The NeurEx led to the strongest COMRISv2 model. Addition of qMRI features enhanced performance only of cognitive disability model, likely because semi-qMRI biomarkers measure infratentorial injury with greater accuracy. COMRISv2 models predict most granular clinical scales in MS with remarkable criterion validity, expanding scientific utilization of cohorts with missing clinical data.

## Introduction

Structural imaging of the central nervous system (CNS) by magnetic resonance (MRI) plays central role in diagnosing multiple sclerosis (MS) and evaluating efficacy of treatments. Nevertheless, the correlations between any MRI biomarker and clinical disability measures are only mild to moderate.

This is explainable by following shortcomings of both clinical scales and MRI biomarkers: A. Reliability: this includes technical aspects of the measurement such as test-retest variability, variability between different raters, different scanners or different analysis methods; and B. Criterion validity: this refers to how each measurement reflects true CNS tissue damage.

While original description of most expert-derived clinical scales missed test-retest reliability (e.g., Expanded Disability Status Scale [EDSS] (1)), the clinical trials identified “transient worsening and improvements” in approximately 20% of subjects (2), likely representing a measurement noise. We developed NeurEx™ App that eliminates part of the noise by algorithmically codified translation of a documented neurological examination into four clinical scales. Although the concordance correlation coefficient (CCC, reflects concordance of two ratings) of neuroimmunology scales between two clinicians transcribing the same documented neurological examination was excellent (i.e., ranging 0.943–0.968; *p*-value < 1 × 10^−7^), the difference for a single exam represented up to 3 EDSS points. By replacing one clinician with the NeurEx™ App that always provides only one rating per scale for a given documented exam, we increased inter-rater reliability to a maximum difference of 1.5 EDSS points (CCC 0.968–0.987; *p*-value <1 × 10^−7^). Of course, NeurEx™ can't eliminate noise stemming from variances in the performance of neurological examination by different clinicians and this likely represents the greater source of noise.

Even more pressing limitation of traditional clinical scales is their sensitivity and construct/criterion validity. For example, natural history cohorts show that on average an MS patient progresses by 1 EDSS point every 10 years (3, 4). Clearly, many axons demyelinate, and oligodendrocytes/neurons die during that time and this ongoing CNS tissue destruction is not captured by EDSS. Additionally, our ability to reliably quantify complex cognitive functions is extremely limited. Consequently, cognitive functions have been severely underrepresented in traditional disability scales. A creative attempt to remedy these limitations was MS functional composite (MSFC), an expert-derived composite scale of three functional tests reflecting ambulation, fine finger movements and memory/processing speed (5). While the concept of MSFC was outstanding, one of the selected components, Paced Auditory Serial Addition Test (PASAT3) proved suboptimal, suffering from high test-retest variability and a learning effect. This limitation was confirmed by developing Combinatorial, weight-adjusted Disability Score (CombiWISE; continuous scale from 0 to 100) (6) using data-driven approach to select contributing features and their optimal “weights”; and neither PASAT3 nor alternative cognitive test Symbol Digit Modalities Test [SDMT (7)] were selected by this model. Even though CombiWISE correlated strongly with EDSS in an independent cohort and measured significant disability progression over 6–12 months, this granular clinical scale still lacks sensitivity to measure destruction of individual axons/neurons and oligodendrocytes, likely happening in MS daily.

As the insensitivity of clinical scales to underlying cellular events is unsurmountable, MRI-based structural imaging and quantification of cellular substructures using advanced imaging methods such as magnetization transfer imaging (MTR) or diffusion tensor imaging (DTI) raised hopes for objective measurements of CNS tissue destruction. Unfortunately, MRI biomarkers proved to have their own limitations. MRI infers CNS structure from the signal decay of energized hydrogen protons, which is dependent on the technical aspects of specific MRI machine and acquisition protocols, on complex post-processing algorithms but also on transient biological processes such as subjects' hydration, use of alcohol or pharmaceutical agents (8). Consequently, test-retest variability of MRI biomarkers is high in comparison to measured change, leading to poor signal-to-noise ratio (SNR). The notable exception are semi-quantitative MRI features (semi-qMRI) such as number of contrast-enhancing lesions or number of (new) T2 lesions formed in different CNS compartments (6, 9, 10), which have excellent SNR.

Additionally, the criterion validity of any single MRI biomarker is problematic as all capture only some aspects of MS-related CNS tissue destruction and do so with restricted specificity. To surpass this limitation, several groups explored combinations of MRI features, akin to composite clinical scales (11–17). All published combinatorial MRI models outperformed each contributing MRI biomarker in correlation with clinical outcomes, validating this concept. Like in combinatorial clinical scales, most groups selected contributing MRI features based on expert opinions (18–21).

We took data driven approach to develop COMRISv1 (Combinatorial MRI scale, version 1), where both selection of contributing features and their weights in the final model were derived from unbiased machine learning (ML) approach (22). COMRISv1 was derived from semi-qMRI features only. This led to high SNR, while, inevitably, sacrificing sensitivity. Despite this, when tested in the independent validation cohort, COMRISv1 models achieved the highest correlations with physical (i.e., EDSS; Rho = 0.7, *p*-value < 0.001, *n* = 114) and cognitive (i.e., SDMT; Rho = 0.5, *p*-value < 0.0001, *n* = 92) disability among all published combinatorial MRI scales for MS. Nevertheless, we wondered, and this paper answers, whether incorporating volumetric qMRI features and using more powerful ML models would strengthen performance of COMRISv2.

## Materials and methods

### Subjects and regulatory approvals

All subjects were prospectively recruited to the National Institute of Allergy and Infectious Diseases (NIAID) of the National Institutes of Health (NIH) natural history protocol “Comprehensive Multimodal Analysis of patients with Neuroimmunological Diseases of the CNS”; Clinicaltrials.gov identifier NCT00794352. The study was approved by NIAID scientific review and by the NIH Institutional Review Board. All subjects provided written informed consent. Supplementary Table S1 contains demographic and clinical data on all subjects.

### Collection and computation of clinical scales

All participants underwent a comprehensive diagnostic process, including neurological examination transcribed to iPad-based App NeurEx™, which automatically calculates four clinical scales, including Expanded Disability Status Scale (EDSS; ordinal scale from 0 to 10) and Scripps Neurological Rating Scale (SNRS, continuous scale from 100 to 0) and streams data to Neuroimmunological Diseases Section (NDS) research database hosted on secured server. Another set of investigators, blinded to NeurEx data collected timed 25-foot walk (25FW), 9-hole peg test (9HPT) and SDMT and inputted these to the NDS database. The database automatically integrates data to calculate CombiWISE. NDS database has also user-defined privileges that blind the clinicians and investigators collecting clinical and functional data to qMRI and semi-qMRI data. MS diagnosis was based on 2010 McDonald diagnostic criteria (23) and, after 2017, based on its 2017 modifications (24).

### Collection and computation of MRI biomarkers

Brain MRIs were performed on Signa – (1.5 T and 3 T, General Electric, Milwaukee, WI) and Skyra – (3 T, Siemens, Malvern, PA) units using 16 – and 32 – channel imaging coils with previously-described scanning protocols (22). Our brain MRI sagittal and axial cuts extend distally to C5 level, allowing determination of semi-qMRI biomarkers of medulla/upper spinal cord (SC) atrophy and lesion load.

The semi-qMRI data were acquired by consensus of MS-trained clinicians during weekly clinical care meetings. The rating of semi-qMRI features was previously extensively described (22) and its codification was integrated to NDS research database.

To acquire qMRI data, T1-magnetization-prepared rapid gradient-echo (MPRAGE) or fast spoiled gradient echo (FSPGR) and T2-weighted three-dimensional fluid attenuation inversion recovery (3D FLAIR) sequences, ideally with 1 mm^3^ isotropic resolution, underwent a five-step pre-processing: (1) de-identification through the elimination of PHI-containing DICOM headers, (2) DICOM to NIFTI transformation, (3) 6-dof alignment to MNI template orientation, using *ANTS* package (25) to first register the T1 image to the 152 MNI template (26) and then co-register the T2 image to the aligned T1 image, (4) SkullStripping the T1 image using *ROBEX* (27) and using the same stripping mask to SkullStrip the co-registered T2 image, and (5) correct bias fields in the T1 image using the N4 algorithm from ANTS (28).

The volumetric data of different CNS structures were then computed by the LesionTOADS algorithm (29) implemented in a cloud based medical image-processing platform, QMENTA as part of collaborative project (https://catalog.qmenta.com/tool/lesion-toads-workflow). LesionTOADS uses an atlas-based technique combining a topological and statistical likelihood atlas for computation of following 12 segmented CNS tissues: Cerebral white matter, Cerebellar white matter, Brainstem, Putamen, Thalamus, Caudate, Cortical gray matter, Cerebellar gray matter, Lesion Volume, Ventricular CSF and Sulcal CSF.

LesionTOADS results were downloaded from QMENTA server and manually quality checked by an investigator blinded to clinical and functional data (MV). 17.2% of scans where LesionTOADS segmentation algorithm masks were incorrectly aligned with targeted anatomical structures were excluded from analyses.

### Development and optimization of COMRISv2 models

COMRISv2 models were constructed using random forest (RF) (30), a decision-tree-based supervised learning algorithm. A decision tree is a modeling approach that uses multiple features (i.e., different MRI-based CNS volumes) to predict an outcome (i.e., disability) by finding the optimal split (e.g., a specific volume) at each branchpoint in the tree. Tree-based models are prone to “overfit” the data. RF aggregates thousands decision trees and uses a random subset of variables for decision-making at each branchpoint to limit (but not eliminate) the overfit problem. Thus, to further optimize our models, we used the iterative process where the least important variable ranked by variable importance function was removed and the RF was rebuilt repeatedly until the root mean square error of the model reached its lowest point. For a visual depiction of this process, see Jackson et al. (31) and Supplementary Figure S1. Default tuning parameters (ntree = 500 and mtry = number of variables/3) were used for all models to ensure fair inter-model comparisons.

### Statistical analyses and implemented safeguards to prevent bias

The correlation between observed and predicted outcomes was assessed by Spearman correlation coefficient Rho. The coefficient of determination (R^2^) measuring the proportion of variance of observed outcomes that is explained by the model prediction, as well as the *p*-value, were calculated from linear regression models. The reproducibility of predicted vs. observed outcomes was evaluated by Lin's concordance correlation coefficient (CCC). The univariate correlations between age, clinical, and MRI outcomes was evaluated using Spearman correlation, the *p*-value cut-off for significant observations was set to 0.001 to account for 31 pairwise comparisons. All statistical analyses were performed in RStudio Version 1.1.463.

The user-defined privileges in the NDS database assured blinding, while software codification of the algorithms for calculating different scales prevented bias in these calculations. Finally, all models were validated in an independent cohort that did not participate in the model development.

## Results

### COMRISv2 model of cognitive disability: SDMT

**Figure 1:**
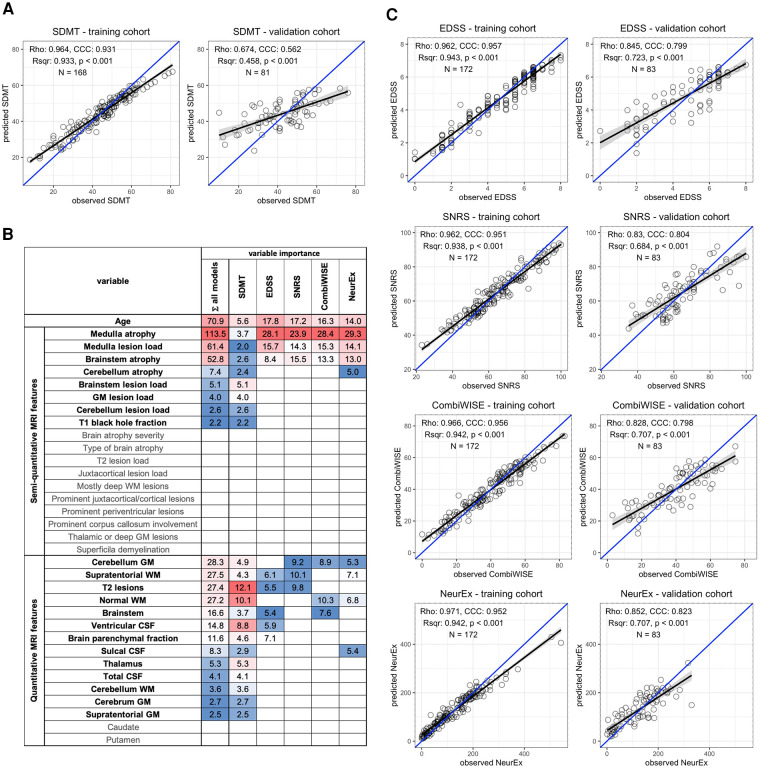
Random forest (RF)-based COMRISv2 models of clinical outcomes. (**A**) RF model of Symbol Digit Modalities Test (SDMT); (**B**) Age, 18 semi-quantitative, and 15 quantitative MRI features were used for RF modelling of the Expanded Disability Status Scale (EDSS), Scripps Neurological Rating scale (SNRS), Combinatorial Weight-adjusted Disability Score (CombiWISE), and digitalized neurological exam score (NeurEx). Variables selected by each model and their respective importance are highlighted here. Variables not selected by any model are depicted in gray. (**C**) RF models of physical disability measured by EDSS, SNRS, CombiWISE, and NeurEx. (**A,C**) The performance of each model was evaluated separately in the training cohort (left plot) and an independent validation cohort (right plot) by plotting observed values on the x-axis and model-predicted values on the y-axis. Spearman Rho, coefficient of determination (Rsqr), *p*-value (*p*), Lin's concordance correlation coefficient (CCC), and number of observations (**N**) are depicted. Black line represents a fitted linear model with the gray-shaded area corresponding to 95% confidence interval. The blue line represents 1:1 fit corresponding to 100% CCC.

The COMRISv2 model optimized to predict SDMT score validated in an independent cohort (Rho = 0.674, *p*-value <0.001, R^2^ = 0.458, CCC = 0.562) (Figure 1A). COMRISv2 SDMT model outperformed the COMRISv1 predictions (Rho = 0.497, *p*-value <0.001, R^2^ = 0.247, CCC = 0.404) of SDMT score in the same cohort. Age and qMRI features ranked most important in the model, although several semi-qMRI features were also included (Figure 1B).

### COMRISv2 models of physical disability: EDSS, SNRS, CombiWISE and NeurEx

COMRISv2 models were also constructed to predict physical disability as measured by four different scales: EDSS, SNRS, CombiWISE, and NeurEx. All models of physical disability performed stronger than the COMRISv2 model for cognitive disability (Figure 1C). The NeurEx scale performed the strongest (Rho = 0.852, *p* value <0.001, R^2^ = 0.707, CCC = 0.823). Models of physical disability favor semi-qMRI biomarkers reflecting disease burden in the infratentorial compartment (Figure 1B).

### Comparing added value of quantitative volumetric features to COMRISv2 models

While semi-qMRI features can be easily collected by any trained clinician or even non-clinical investigator with knowledge of brain/spinal cord anatomy, collection of qMRI features require more specialized skillset and much more resources. To facilitate decisions about resource allocation, we formally assessed value of semi-qMRI and qMRI features for predicting different disability outcomes.

Thus, COMRISv2 models for SDMT were constructed considering only qMRI measures or only semi-qMRI measures, both in presence and absence of age. Cognitive disability models considering only qMRI measures (Rho = 0.568, *p*-value <0.001, R^2^ = 0.363, CCC = 0.497) performed slightly better than those considering only semi-qMRI measures (Rho = 0.544, *p*-value <0.001, R^2^ = 0.282, CCC = 0.474), but none outperformed the original model that integrates qMRI, semi-qMRI, and age (Rho = 0.674, *p*-value <0.001, R^2^ = 0.458, CCC = 0.562).

**Figure 2:**
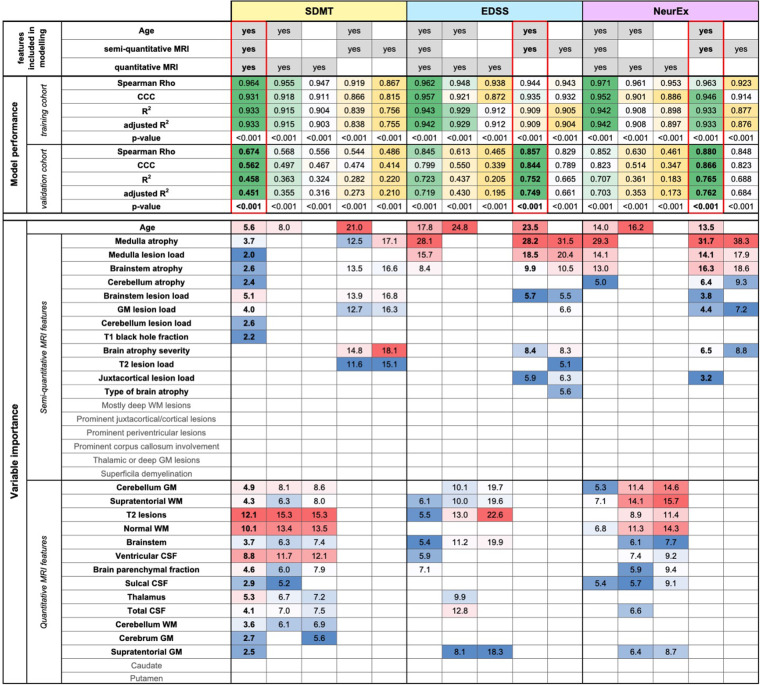
Evaluation of added value of quantitative MRI features. The original random forest (RF) models used age, semi-quantitative, and quantitative MRI features as an input. We tested how the RF models of Symbol Digit Modalities Test (SDMT), Expanded Disability Status Scale (EDSS), and digitalized neurological exam score (NeurEx) would perform if only semi-quantitative or only quantitative MRI features (with or without age) would perform. The performance of each model was evaluated separately in the training and an independent validation cohort by calculating Spearman Rho, Lin's concordance correlation coefficient (CCC), coefficient of determination (R^2^), adjusted coefficient of determination (adjusted R^2^), and *p*-value. The best performing models are highlighted by red rectangles. Variables selected by each model and their respective importance is also depicted.

When we performed the same comparison in physical disability models (NeurEx and EDSS), addition of qMRI features did not improve model performance. NeurEx and EDSS models that considered only age and semi-qMRI features outperformed models that included qMRI features (Figure 2, Supplementary Figure S2).

### Comparing feature selection between different COMRISv2 models with univariate correlations between MRI biomarkers and clinical outcomes

**Figure 3:**
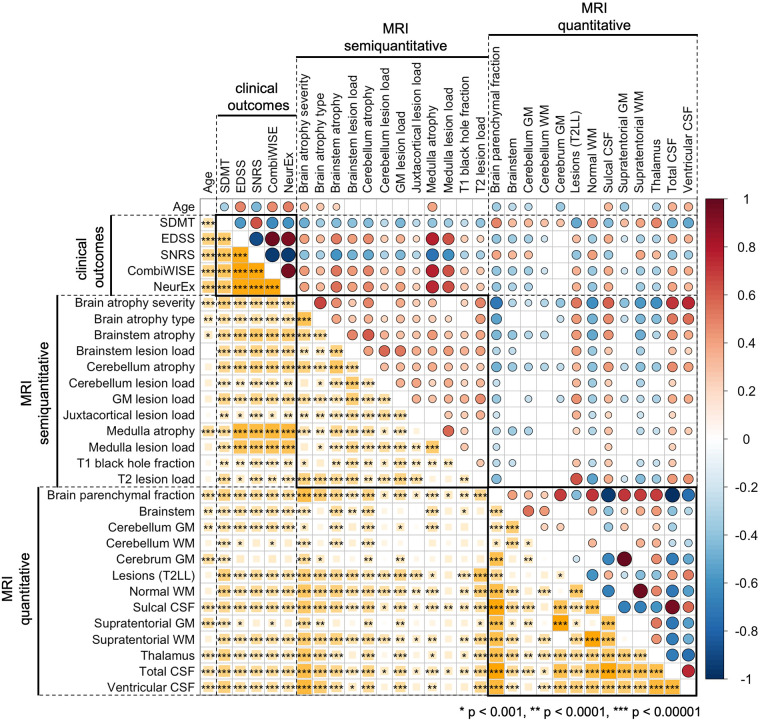
Univariate correlations between age, clinical outcomes, semiquantitative, and quantitative MRI features. Correlations were evaluated using Spearman Rho, with positive correlation in shades of red and negative correlations in shades of blue. The size of the circles above the diagonal and the size of the yellow squares below the diagonal corresponds to the absolute value of Rho. Only correlations with *p*-value below 0.001 were considered significant (accounting for 31 comparisons performed). The number of stars corresponds to the level of statistical significance.

To facilitate interpretability of COMRIS models, we examined univariate correlations between all MRI features selected by at least one COMRIS model and all clinical outcomes plus age (Figure 3).

As would be expected, all clinical outcomes correlated moderately with age. qMRI outcomes related to CSF and Brain parenchymal fraction also correlated with age. From infratentorial structures, only cerebellar gray matter (GM) and semi-qMRI biomarker of brainstem and medulla/upper spinal cord (SC) atrophy correlated with age.

Most qMRI measures correlated with each other, except cerebellar GM, the only infratentorial qMRI biomarker selected by four out of five COMRIS models, which showed only weak correlations with few outcomes. All semi-qMRI biomarkers correlated with each other, but the correlations were generally weak to moderate. Semi-qMRI features also correlated with qMRI features, except medulla/upper SC lesion load that correlated marginally with sulcal CSF. Although medulla/upper SC atrophy correlated with most of qMRI outcomes, these correlations were marginal with qMRI volumetric measures of telencephalon and strongest for brainstem and cerebellum GM volume. Thus, we conclude that qMRI and semi-qMRI measures provide mostly complimentary information, with qMRI outcomes better reflecting telencephalon tissue damage and semi-qMRI outcomes better capturing infratentorial tissue damage.

All clinical outcomes correlated with each other, with SDMT exhibiting only moderate correlations, while other clinical scales correlated strongly. The difference between SDMT and all remaining clinical outcomes was also evident from correlations with MRI outcomes: qMRI biomarkers correlated stronger with SDMT (cognitive disability) in comparison to clinical outcomes that capture predominantly physical disability. In contrast, semi-qMRI biomarkers, especially medulla/upper SC atrophy and lesion load, followed by brainstem atrophy, correlated with non-SDMT clinical outcomes and these correlations were overall stronger than correlations of qMRI measures with SDMT. All qMRI biomarkers correlated with physical disability outcomes (i.e., EDSS, SNRS, CombiWISE and NeurEx) weaker than age, whereas many qMRI outcomes outperformed age in correlation with SDMT.

## Discussion

With the technological advances that allow reliable measurements of genetic, transcriptomic and proteomic biomarkers in hundreds of patients, the data scientists are realizing that the most limiting obstacle in translating these “omics” data into clinically translatable insights are, surprisingly, poor quality clinical and imaging data. This sentiment is epitomized in the recent review: “It is amazing how bad the standard data sets in the medical domain are (noisy, sparse, wrong, biased, etc).” (32) Employing unbiased, data-driven approaches to develop more accurate tools for measuring neurological disability and CNS tissue damage and validating both their criterion validity and reproducibility is the way to transcend this conundrum.

This paper demonstrates the power of ML approach to assemble semi-qMRI and qMRI brain imaging biomarkers into combinatorial models (COMRISv2) that reliably predict neurological disability in MS patients. Compared to previously published composite MRI scales, our study has following strengths: (1) The MRI features and their weights are selected using unbiased, data-driven approach; (2) We studied a moderately large cohort of MS patients, with good representation of subjects with progressive MS; (3) COMRISv2 tested both semi-qMRI and qMRI volumetric data; (4) In addition to EDSS, we modeled COMRISv2 predictions of physical disability against SNRS, highly granular CombiWISE and NeurEx scales, and predictions of cognitive disability against SDMT; (5) Our models are validated in the independent cohort of MS patients that did not contribute to the development or optimization of the model(s).

We also recognize the following limitations of current study: (1) Although our original COMRISv1 computation is publicly available, including detailed guideline for semi-qMRI ratings (22), and we observed that adherence to those guidelines leads to mean interrater variability less than 10%, up till now no external group attempted to reproduce our data. This causes uncertainty whether other investigators could achieve analogous reproducibility of COMRIS models; (2) We did not test qMRI measures of atrophy or T2LL in the medulla/upper SC, as Lesion-TOADS algorithm does not provide these outcomes and also because we lacked dedicated SC MRI; (3) Our study did not include qMRI biomarkers derived from advanced imaging methods such as MTR or DTI.

While we can't influence the first limitation, we can address the effect of subsequent two limitations by literature review: First, high quality volumetric SC data require dedicated SC imaging, not available in our patients. Second, published observations suggest that addition of qMRI cervical SC biomarkers would have limited effect on COMRISv2 performance: e.g., the second iteration of Magnetic Resonance Disease Severity Scale (MRDSS2) (21) demonstrated that addition of upper cervical SC area to MRDSS1 model (which consisted only of brain qMRI features) increased correlation with EDSS from 0.25 to 0.33 (*p* = 0.013). Both COMRISv1 and COMRISv2 models (using only semi-qMRI features) validated much stronger correlations with EDSS (i.e., Rho = 0.857, *p* < 0.001). Analogously, meta-analysis (21 studies/1,933 participants) of dedicated 3 T SC imaging showed moderate correlation of cervical SC atrophy with EDSS (Rho = −0.42; *p* < 0.0001) (33). This likely represents over-estimation, as included studies with small number of participants showed invariably larger correlations. It has been convincingly shown that small studies over-estimate effect size (34). Correspondingly, large study (*n* = 1,249) published after the aforementioned meta-analysis measured Rho −0.315 (*p* < 0.01) for correlation of cervical SC volume with EDSS (35). These are analogous or smaller univariate correlations as those we observed in COMRIS models between EDSS and two highest ranking semi-qMRI biomarkers: medulla/cervical SC atrophy and T2LL [Figure 3 and (22)].

Based on the limited value of volumetric qMRI compared to semi-qMRI biomarkers in COMRISv2 models of physical disability, we do not expect that incorporating MTR or DTI data could meaningfully enhance correlations with clinical outcomes for several reasons: (1) These advanced imaging biomarkers have even poorer SNR than volumetric qMRI measures (6, 10); and (2) COMRISv2 optimized for CombiWISE or NeurEx already explains close to 70% of physical disability variance in the independent validation cohort, which is exceptionally good performance.

To put our results into perspective, we performed a meta-analysis of 302 studies describing ML models of MS disability and severity outcomes (36), including 40 studies that modeled EDSS as an ordinal scale. Only half of those reported effect sizes. The meta-analysis evaluated published studies based on seven criteria (e.g., blinding, outlier removal, explanation of missingness, adjustment for confounders, adjustment for multiple comparison, presence of controls, and validation) and identified significant negative correlation between effects size and number of criteria fulfilled. An independent validation cohort, used in our study, that is essential to understand the true predictive power of composite construct on patients whose data did not contribute to model development, was missing in all published MRI studies predicting EDSS. Only one study out of 20 showed cross-validation results for EDSS models, achieving R^2^ of 0.16–0.19 (37). In comparison, our optimized EDSS model explains 75% of variance in the independent validation cohort. Similar observation was made for MRI biomarker-based models of SDMT – 5 out of 12 studies reported effect sizes as R^2^ ranging from 0.3 to 0.62 in the training cohort, compared to our optimized SDMT model that reaches R^2^ of 0.933 in the training and 0.46 in the independent validation cohort. Presented data, congruent with most independent validation studies published, show unequivocally that training cohort results always over-estimate true strength of the relationships. Thus, we conclude that COMRISv2 models achieve the highest effect sizes in predicting clinical disability outcomes among published studies.

The limitation of the criterion validity of simple volumetric qMRI biomarkers we mentioned in the introduction is exemplified in highly informative post-mortem imaging pathological assessment, which showed that SC atrophy (19%–24%) strongly under-estimates axonal loss (57%–62%) in MS (38). Because these imaging data were postmortem, they were not affected by motion artifacts and signal averaging which would further decrease the strength of relationship between imaging biomarker and histologically measured CNS tissue destruction. Therefore, at very best technical imaging conditions the criterion validity of volumetric qMRI biomarkers of SC is limited.

Nevertheless, qMRI biomarkers, especially when measuring large telencephalon structures have validated relationship to CNS tissue destruction in MS: (1) Brain atrophy is higher in MS compared to HV; (2) It correlates with disability in large cohorts and (3) It predicts disability progression in long longitudinal studies (39–41). Consequently, if we could measure volume of all CNS structures with high accuracy, qMRI biomarkers would likely outperform semi-qMRI biomarkers in all models, as we observed for SDMT version of COMRISv2.

Unfortunately, the test-retest variance (“noise”) of qMRI outcomes increases inversely to the size and contrast of the structure measured and the required scanning time. Motion artifacts from skeletal muscles, heartbeats, breathing and cerebrospinal fluid pulsation disadvantage qMRI biomarkers from infratentorial structures compared to large telencephalic structures. Thus, volumetric biomarkers derived from small infratentorial structures with low MRI contrast from neighboring tissues, that need long acquisition times will have high “noise” (and low SNR) (6). This explains why infratentorial semi-qMRI biomarkers, while theoretically less sensitive, outperformed infratentorial qMRI features (Figures 1B, 2): because they are measured with higher SNR. Research advances to limit measurement noise of infratentorial qMRI biomarkers may have greater clinical value than development of imaging methods that require longer scanning times and increased complexity of mathematical/physical data manipulations to produce quantitative output.

In conclusion, this study demonstrates excellent criterion validity of measuring CNS tissue damage in MS by two different modalities: neurological examination and brain MRI. There is nothing in clinical data that makes them inevitably poor quality (i.e., noisy, sparse, wrong, biased) if we approach their collection and their aggregation into sensitive and accurate scales with the same scientific rigor used to optimize collection and quantification of omics data. The observations that novel scales of neurological disability with much broader dynamic range than EDSS (i.e., total of 20 possible disability progression steps in the ordinal EDSS scale, vs. practically unlimited range of CombiWISE [0–100 continuous scale] and NeurEx [0 to theoretical maximum of 1,349] values) validated comparably, or even outperformed EDSS demonstrates that implementing data-driven approaches to development of new clinical scales allows increasing sensitivity without limiting their accuracy. The CCC of 0.866 between semi-qMRI features-derived COMRISv2 and NeurEx in the independent validation cohort indicates that scientists have at their fingertips a reliable inexpensive tool that can predict the most granular scale of neurological disability we currently have in MS research.

## Data Availability

The original contributions presented in the study are included in the article/**Supplementary Material**, further inquiries can be directed to the corresponding author/s.
