# Supplementary material for: Machine learning-optimized Combinatorial MRI scale (COMRISv2) correlates highly with cognitive and physical disability scales in Multiple Sclerosis patients
Source: Front Radiol. 2022 Nov 11;2:1026442. doi: 10.3389/fradi.2022.1026442 (PMC10365117; doi:10.3389/fradi.2022.1026442)

Supplemental table 1: Demographic data

	training dataset	*validation dataset*
**N (% F)**	172 (52.9)	83 (59.0)
**% RRMS/SPMS/PPMS/CIS-RIS**	41.3/22.1/33.7/2.9	34.9/28.9/31.3/4.8
**Age (mean, SD)**	53.2 (12.3)	53.0 (12.1)
**SDMT (mean, SD)**	44.1 (14.2)	42.7 (13.7)
**EDSS (mean, SD)**	4.8 (1.9)	4.8 (1.9)
**SNRS (mean, SD)**	64.9 (16.4)	64.5 (14.8)
**CombiWISE (mean, SD)**	39.4 (17.6)	39.5 (16.5)
**NeurEx (mean, SD)**	127.7 (93.4)	126.1 (80.5)

<u>Supplemental Figure 1</u>: The below plot depicts the model optimization process. Models were re-constructed with the least important variable removed until the root mean square error reached its lowest point and increased. The red line shows the point where the root mean square error is lowest; this is the number of variables included in the optimized model.

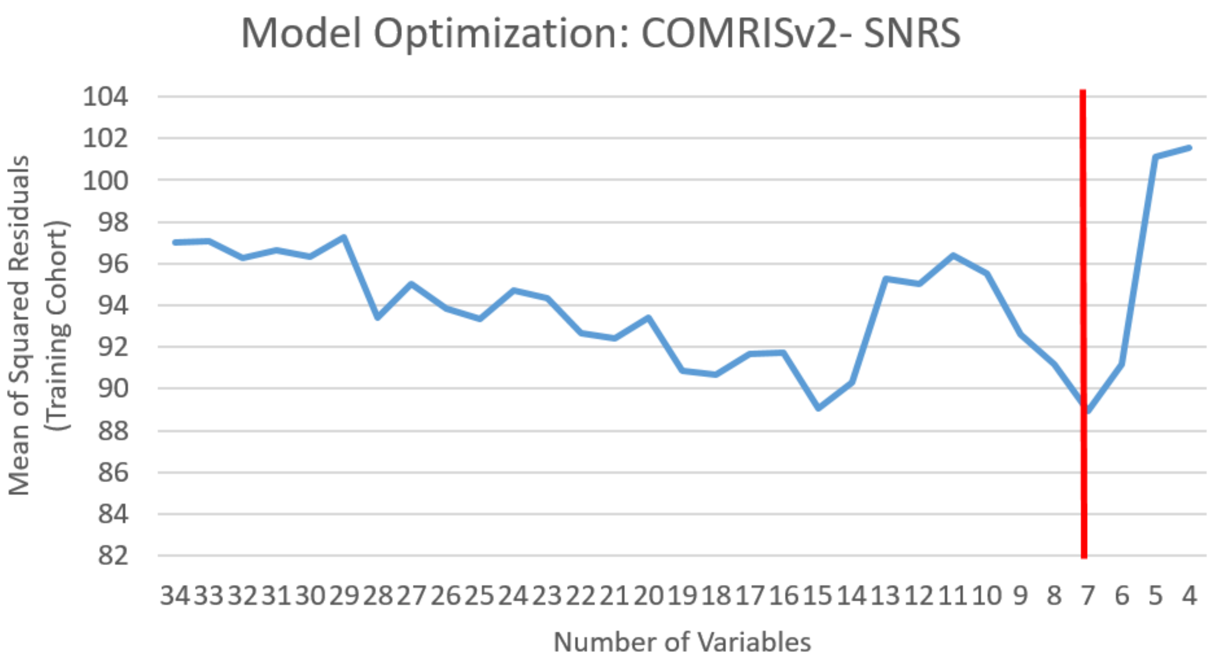

Supplemental Figure 2: Scatter plots of measured vs predicted outcomes from various RF models of Symbol Digit Modalities Test (SDMT), Expanded Disability Status Scale (EDSS), and digitalized neurological exam score (NeurEx) using all predictors, fully quantitative predictors + age, fully quantitative predictors without age, semi-quantitative predictors with age, and semi-quantitative predictors without age. The performance of each model was evaluated separately in the training and an independent validation cohort by calculating Spearman Rho, Lin's concordance correlation coefficient (CCC), coefficient of determination (Rsqr), and p-value. Blue lines represent 1:1 line, black line represents linear model, gray shaded area represents confidence interval.

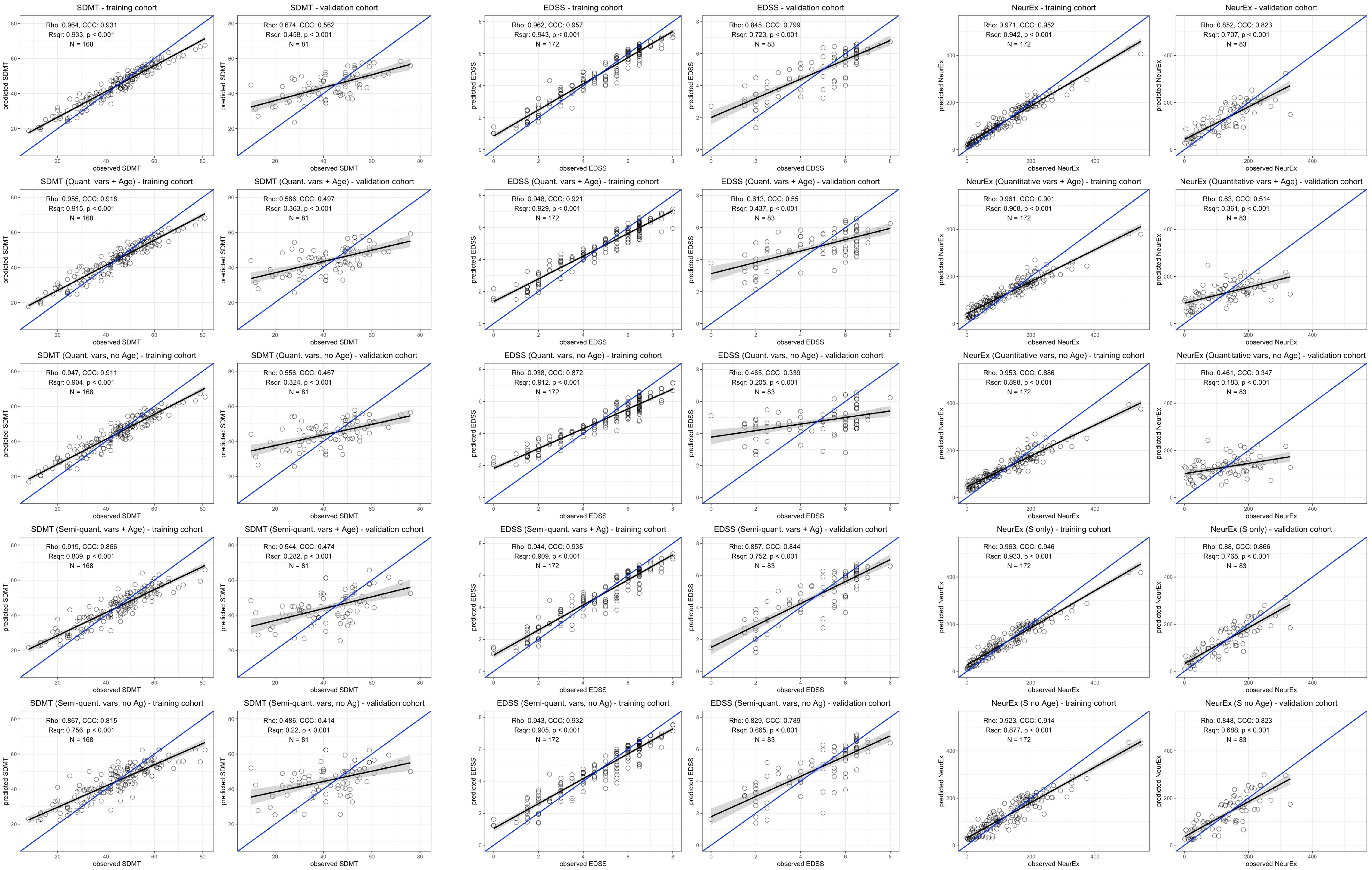

Supplement: Supplementary file 2 [file Datasheet1.pdf]
